# Association between apolipoprotein B genetic polymorphism and the risk of calcific aortic stenosis in Chinese subjects, in Xinjiang, China

**DOI:** 10.1186/s12944-018-0696-6

**Published:** 2018-03-07

**Authors:** Yong-Tao Wang, Yang Li, Yi-Tong Ma, Yi-Ning Yang, Xiang Ma, Xiao-Mei Li, Fen Liu, Bang-Dang Chen

**Affiliations:** 1 Department of Cardiology, First Affiliated Hospital of Xinjiang Medical University, Urumqi, 830054 People’s Republic of China; 2 Xinjiang Key Laboratory of Cardiovascular Disease Research, Urumqi, 830054 People’s Republic of China

**Keywords:** apoB, Calcific aortic stenosis, Single nucleotide polymorphism

## Abstract

**Background:**

Limited information is available when it comes to the impact of genetic on Calcific Aortic Stenosis (CAS). Apolipoprotein B (apoB) is a key component in lipid metabolism and plays an important role in the dynamic equilibrium of cholesterol. We performed a case–control study to explore the association of apoB genetic polymorphisms with CAS in Chinese subjects, in Xinjiang, China.

**Methods:**

We designed a case-control study including 314 CAS patients and 652 age- and sex-matched control subjects. Using the polymerase chain reaction-restriction fragment length (PCR-RFLP) method, we genotyped two SNPs (rs6725189 and rs693) of apoB gene in all subjects.

**Results:**

We found that the rs693 T allele was associated with a significantly elevated CAS risk [TT/CT vs. CC: adjusted odds ratio (AOR) = 1.58, 95% confidence interval (CI) = 1.82–2.10, *P* = 0.002] and the rs6725189 T allele was also associated with a significantly elevated CAS risk (GT vs. GG: AOR = 1.82, 95% CI = 1.14–2.92, *P* = 0.013). Furthermore, we also found that the TC levels were significantly higher in rs693 TT/CT genotypes than that in CC genotypes (*P* < 0.05).

**Conclusions:**

Both rs693 and rs6725189 of the apoB gene are associated with CAS in Chinese subjects, in Xinjiang, China.

## Background

With the increasing of elderly population and improvement of living standards, calcified valvular heart disease has outpaced rheumatic valvular disease to become the most common heart valve disease [1]. Calcific aortic stenosis (CAS), as the most common type of calcified valvular heart disease, is an important cause of increased morbidity and mortality [2–4]. The prevalence of CAS in adults older than 75 years is higher than 3% and the figure may double within the next 50 years [5, 6]. Furthermore, CAS is also the most common indication for surgical valve replacement and transcatheter aortic valve implantation [7, 8]. The high prevalence of CAS, together with its severe prognosis reveal it to be an increasing burden.

Apolipoprotein B (apoB) is a key structural component of all the atherogenic lipoproteins (LDL, very low-density lipoprotein (VLDL), intermediate-density lipoprotein (IDL) and lipoprotein a) and plays an important role in the dynamic equilibrium of cholesterol [9]. The Copenhagen City Heart Study is the first prospective study to estimate the predictive role of apoB in ischemic cardiovascular disease risk in the general population in both women and men. The results from this study showed that apoB was superior to LDL-C in the prediction of cardiovascular disease [10].

The apoB gene is located on the short arm of chromosome 2 and spans 43 kilobases, including 28 exons. Studies performed on twins indicated that genetic components account for 50–60% of the variation in plasma apoB levels [11]. In addition, several other studies have demonstrated that polymorphisms of the apoB gene are associated with atherosclerosis [12–14]. However, the association between apoB gene and CAS was still unclear. The present case-control study aimed to explore the association of apoB gene polymorphisms with CAS in Chinese subjects.

## Methods

### Ethics statement

This study was approved by the Ethics Committee of the First Affiliated Hospital of Xinjiang Medical University (Xinjiang, China). It was conducted according to the standards of the Declaration of Helsinki. Written informed consent was obtained from each participant, who explicitly provided permission for all DNA analyses and the collection of relevant clinical data.

### Subjects

All of the participants were Han Chinese and were selected from the First Affiliated Hospital of Xinjiang Medical University. The time period of the study was from January 2012 to January 2016. A total of 314 subjects diagnosed with CAS and 652 health controls were recruited. CAS was defined as thickened and/or calcified aortic leaflets with restricted systolic motion, a calculated aortic valve area 1.8 cm^2^ and a transaortic mean pressure gradient 10 mmHg [15, 16]. Subjects who did not have relevant valvular abnormalities in echocardiograms or a history of valvular heart disease were selected as the control group. Subjects with rheumatic heart disease, congenital heart disease, chronic kidney disease, and syphilitic heart disease were excluded. Hypertension was defined as self-reported use of antihypertensive medication within the past 2 weeks or an average systolic blood pressure ≥ 140 mmHg, an average diastolic blood pressure ≥ 90 mmHg, or both. Diabetes was defined as fasting plasma glucose ≥6.99 mmol/L, the use of insulin or oral hypoglycemic agents, or a self-reported history of diabetes. Smoking was defined as currently smoking cigarettes. We have added these to the method section.

### Biochemical analyses

Blood samples were obtained from an antecubital vein into vacutainer tubes containing EDTA in the morning after an overnight fasting period. We measured the plasma concentration of blood triglyceride (TG), total cholesterol (TC), low density lipoprotein (LDL), high density lipoprotein (HDL) and fasting plasma glucose (FPG) in the Clinical Laboratory Department of the First Affiliated Hospital of Xinjiang Medical University [17, 18].

### APOB genotyping

**Fig. 1:**
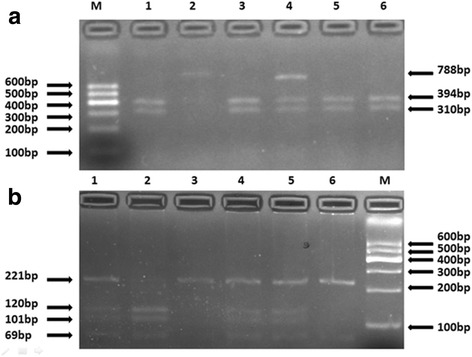
The restriction fragment length polymorphism analysis to determine the genotype. **a**. For rs6725189, the TT genotype shows two bands at 788 bp and 84 bp (2); the GG genotype shows three bands at 394 bp, 310 bp and 84 bp (1, 3, 5 and 6); and the GT genotype shows four bands at 788 bp, 394 bp, 310 bp and 84 bp (4). **b**. For rs693, the CC genotype shows two bands at 221 bp and 69 bp (3 and 6); the TT genotype shows three bands at 120 bp, 101 bp and 69 bp (2); and the CT genotype shows four bands at 221 bp, 120 bp, 101 bp and 69 bp (1, 4 and 5)

We selected two single-nucleotide polymorphisms (SNPs) in the human APOB gene as markers for assessment of genetic association. There are 5453 SNPs for the human apoB gene listed in the National Center for Biotechnology Information SNP database (http://www.ncbi.nlm.nih.gov/SNP). Using Haploview 4.2 software and International HapMap Project website phase I&II data base (http://www.hapmap.org), we obtained two tagging SNPs (rs6725189,rs693) using minor allele frequency (MAF) ≥0.05 and linkage disequilibrium patterns with r2 ≥ 0.8 as a cutoff. Genomic DNA was extracted from peripheral blood leukocytes using a DNA extraction Kit (Beijing Bioteke Company Limited, Beijing, China). Genotyping in this present study was confirmed via polymerase chain reaction (PCR)-restriction fragment length polymorphism (RFLP) analysis. Sequencing primers were designed using Primer Premier 5.0 software. Synthesis of the Premier was undertaken by Shanghai Genery Biological Technology Company Limited (Shanghai, China). PCR amplification was performed using 25 μL of 2*powder Taq PCR master mix (Beijing Biotech, Beijing, China), 50 ng of genomic DNA, 21 μL of distilled water, 1 μL of each forward and reverse primer in a 50 μL final reaction volume. The thermal cycling conditions were as follows: an initial denaturation step at 95 °C for 5 min; 35 cycles of 95 °C for 30 s, 51.3 °C for 30 s and 72 °C for 45 s was followed by a final extension step of 72 °C for 10 min. Thermal cycling was performed using the GeneAmp 9700 system (Applied Biosystems). Digestion of PCR products by restriction enzymes was conducted according to the manufacturer’s instructions [19]. The primer pair sequences, annealing temperatures, resulting fragments and restriction enzymes for the two SNPs are detailed in Table 1. The digested products were analyzed on 3% agarose gels and stained with ethidium bromide (Fig. 1). In our test, there exist at least one positive and one negative control per 96-well DNA plate and we also duplicated 10% of the total genotyped samples. The rate of genotyping success for each SNP was 100%.

**Table 1:** The primer sequences for each SNP

SNPs	Polymerase chain reaction primers	Denaturation temperature	Product length	Restriction enzyme
Rs6725189	Sense 5′TATTCCCTATTATGTTGTGG3′	51.3 °C	788	Ace I
	Antisense 5′CTTGAAGGTGGACTGGTT 3′			
Rs693	Sense 5′GGAAAGCCTACAGGACAC3′	51.3 °C	290	FnuA I
	Antisense 5′TCATACGTTTAGCCCAAT3′

### Statistical analysis

Data analysis was performed using SPSS version 17.0 for Windows (SPSS Inc., Chicago, IL, USA). Hardy-Weinberg equilibrium was assessed via chi-square analysis. Measurement data are shown as the mean ± SD, and the differences between CAS and control subjects were assessed using independent-sample t-test. Differences in enumeration data such as frequencies of smoking, drinking, hypertension and apoB genotypes between the CAS and control subjects were analyzed using the chi-square test. Additionally, logistic regression analyses with effect ratios (odds ratio [OR] and 95% CI) were used to assess contribution of major risk factors. *P* value < 0.05 was considered statistically significant.

## Result

### Characteristics of subjects

The baseline characteristics of 314 CAS patients and 652 control subjects were shown in Table 2. The mean age, male to female ratio, BMI, TG and HDL-C levels were similar between the controls and CAS patients (all *P* > 0.05). The SBP, DBP, glucose, TC, LDL-C levels and the percentages of subjects who have hypertension, diabetes and smoke were significantly higher in the CAS patients than in the controls (all *P* < 0.05).

**Table 2:** Clinical and metabolic characteristics of subjects

Characteristic	Control(*n* = 652)	Case(*n* = 314)	*P* value
Age(years)	67.69 ± 8.85	67.39 ± 9.76	0.633
Male/female	360/292	158/156	0.153
BMI(kg/m^2^)	25.91 ± 3.90	26.17 ± 4.13	0.344
SBP(mmHg)	141.25 ± 20.83	149.69 ± 25.64	< 0.001
DBP(mmHg)	86.49 ± 16.25	92.78 ± 20.86	< 0.001
Hypertension(%)	333(51.1)	213(67.8)	< 0.001
Diabetes(%)	50(7.7)	41(13.1)	0.007
Smoking(%)	141(21.6)	98(31.2)	0.001
Glucose(mmol/L)	5.21 ± 1.56	5.51 ± 2.02	0.011
TC(mmol/L)	4.60 ± 1.11	4.84 ± 1.14	0.002
TG(mmol/L)	1.52 ± 1.15	1.51 ± 1.05	0.915
LDL-C(mmol/L)	2.71 ± 0.88	2.97 ± 0.99	< 0.001
HDL-C(mmol/L)	1.26 ± 0.49	1.26 ± 0.45	0.939

Continuous variables are expressed as mean ± SD. Categori cal variables are expressed as percentag es

The *P* value of the continuous variables was calculated by the independent samples t test. The *P* value of the categorical variables was calculated by χ2 test

*TG* triglyceride, *TC* total cholesterol, *HDL-C* high density lipoprotein-cholesterol, *LDL-C* low density lipoprotein-cholesterol

### Distributions of genotype and allele in patients with valvular calcification and controls

Table 3 shows the distribution of genotypes and alleles for the two SNPs (rs6725189 and rs693) of the apoB gene. The genotype distributions of the two SNPs were in accordance with the Hardy-Weinberg equilibrium in controls (all *P* > 0.05). For rs693, the distribution of the genotypes, the dominant model (TT + CT vs CC), the additive model (CT vs CC + TT) and the allele frequency showed significant differences between the controls and CAS patients(*P* = 0.004, *P* = 0.001, *P* = 0.015, *P* <0.001, respectively). For rs6725189, the distribution of the genotypes showed significant differences between the controls and CAS patients (*P* = 0.044).

**Table 3:** Distribution of SNPs of apoB gene in CAS and controls

Genotype or allele	Control[n(%)]	CAS[n(%)]	*P* value
rs693			
CC	430(66.0)	172(54.8)	
CT	150(23.0)	95(30.2)	
TT	72(11.0)	47(15.0)	0.004
Dominant			
TT + CT	222(34.0)	142(45.2)	
CC	430(66.0)	172(54.8)	0.001
Recessive			
TT	72(11.0)	47(15.0)	
CC + CT	580(89.0)	267(85.0)	0.082
Additive			
CT	150(23.0)	95(30.2)	
CC + TT	502(77.0)	219(69.8)	0.015
Allele			
C	1010(77.5)	439(69.9)	
T	294(22.5)	189(30.1)	< 0.001
rs6725189			
GG	589(90.3)	270(86.0)	
GT	63(9.7)	44(14.0)	0.044
Allele			
G	1241(95.2)	584(93.0)	
T	63(4.8)	44(7.0)	0.051

### Stratified analysis between apoB gene polymorphisms and CAS risk

We performed stratifcation analyses in terms of gender, hypertension, diabetes and smoking status to evaluate how these variables modifed the association between the SNPs (rs693 and rs6725189) and CAS risk (Table 4). Multiple logistic regression were used to adjust confounding factors. For female, the dominant model (TT + CT vs CC) of rs693 remain significantly associated with CAS (OR = 1.89, 95% CI = 1.27–2.81, *P* = 0.002). For male and female, rs6725189 remain significantly associated with CAS (male: OR = 1.59, 95% CI = 1.04–2.43, *P* = 0.032; female: OR =1.69, 95% CI = 1.14–2.52, *P* = 0.010). For subgroup with hypertension, the dominant model (TT + CT vs CC) of rs693 remain significantly associated with CAS (OR = 1.54, 95% CI = 1.07–2.23, *P* = 0.020). For subgroup with and without hypertension, rs6725189 remain significantly associated with CAS (subgroup with hypertension: OR = 1.57, 95% CI = 1.09–2.27, *P* = 0.015; subgroup without hypertension: OR =1.70, 95% CI = 1.05–2.76, *P* = 0.031). For subgroup without diabetes, the dominant model (TT + CT vs CC) of rs693 remain significantly associated with CAS (OR = 1.52, 95% CI = 1.12–2.06, *P* = 0.007). For subgroup with diabetes, rs6725189 remain significantly associated with CAS (OR = 1.67, 95% CI = 1.23–2.26, *P* = 0.001). For nonsmoker and smoker, the dominant model (TT + CT vs CC) of rs693 and rs6725189 remain significantly associated with CAS(rs693: nonsmoker: OR = 1.41, 95% CI = 1.01–1.97, *P* = 0.046, smoker: OR = 2.16, 95% CI = 1.21–3.88, *P* = 0.010; rs6715189: nonsmoker: OR = 1.44, 95% CI = 1.03–2.02, *P* = 0.032, smoker: OR = 2.51, 95% CI = 1.39–4.53, *P* = 0.002).

**Table 4:** Stratifed analysis between apoB gene polymorphisms and CAS risk

	Case/control	rs693 (Genetype)	Adjusted OR (95% CI)	*P* value	rs6725189 (Genetype)	Adjusted OR (95% CI)	*P* value
Gender							
Male	158/360	TT + CT/CC	1.295(0.848–1.977)	0.232	GT/GG	1.592(1.042–2.433)	0.032
Female	156/292	TT + CT/CC	1.888(1.266–2.814)	0.002	GT/GG	1.689(1.135–2.515)	0.01
Hypertension							
No	101/319	TT + CT/CC	1.563(0.961–2.541)	0.072	GT/GG	1.701(1.049–2.757)	0.031
Yes	213/333	TT + CT/CC	1.544(1.071–2.226)	0.02	GT/GG	1.574(1.092–2.271)	0.015
Diabetes							
No	273/602	TT + CT/CC	1.521(1.121–2.064)	0.007	GT/GG	1.668(1.230–2.263)	0.001
Yes	41/50	TT + CT/CC	1.854(0.714–4.814)	0.205	GT/GG	1.221(0.474–3.143)	0.679
Smoking							
Nonsmoker	216/511	TT + CT/CC	1.407(1.005–1.969)	0.046	GT/GG	1.444(1.032–2.021)	0.032
Smoker	98/141	TT + CT/CC	2.163(1.206–3.877)	0.01	GT/GG	2.509(1.390–4.527)	0.002

### Related risk factors for CAS

Multivariate logistic analysis was used to assess contribution of major risk factors in the whole sample which contains all 966 participants. Multiple logistic regression showed that the incidence of CAS positively correlated with hypertension, diabetes, smoking, TC, LDL-C, rs693 TT/CT genotypes and rs6725189 GT genotypes (Tables 5 and 6).

**Table 5:** Results of Logistic analysis (rs6725189)

Variables	OR	95% CI	*P* value
rs6725189(GT vs.GG)	1.819	1.135–2.916	0.013
Gender	1.023	0.740–1.416	0.888
Age	0.994	0.978–1.009	0.425
BMI	0.988	0.953–1.025	0.536
Hypertension	2.294	1.683–3.127	< 0.001
TG	0.923	0.802–1.063	0.266
TC	1.21	1.061–1.380	0.005
HDL-C	0.855	0.623–1.174	0.333
LDL-C	1.393	1.192–1.628	< 0.001
Diabetes	1.514	0.923–2.483	0.1
Smoking	1.822	1.268–2.618	0.001

**Table 6:** Results of Logistic analysis (rs693)

Variables	OR	95% CI	*P* value
rs693(CT + TT vs. CC)	1.577	1.182–2.103	0.002
Gender	0.998	0.722–1.379	0.989
Age	0.995	0.980–1.011	0.576
BMI	0.987	0.952–1.024	0.495
Hypertension	2.147	1.583–2.911	< 0.001
TG	0.928	0.806–1.069	0.301
TC	1.191	1.044–1.359	0.009
HDL-C	0.872	0.635–1.198	0.399
LDL-C	1.387	1.187–1.621	< 0.001
Diabetes	1.846	1.155–2.950	0.01
Smoking	1.798	1.252–2.581	0.001

### Genotypes and serum lipid levels

**Fig. 2:**
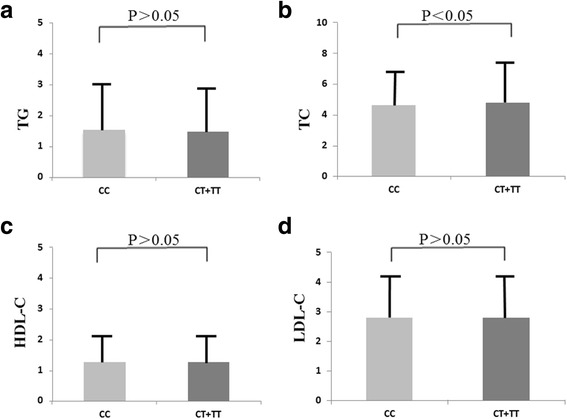
Association between rs693 and lipid parameters. **a**. There exists no significant difference of TG between rs693 TT/CT genotypes and CC genotypes (*P* > 0.05); **b**. The TC levels were significantly higher in rs693 TT/CT genotypes than that in CC genotypes (*P* < 0.05); **c**. There exists no significant difference of HDL-C between rs693 TT/CT genotypes and CC genotypes (*P* > 0.05); **d**. There exists no significant difference of LDL-between rs693 TT/CT genotypes and CC genotypes (*P* > 0.05)

As shown in Fig. 2, the TC levels were significantly higher in rs693 TT/CT genotypes than that in CC genotypes (*P* < 0.05).

## Discussion

In this investigation of apoB alleles and CAS, we found that variation in the apoB gene is associated with CAS. To date, this is the first study to investigate the association between the human apoB gene and CAS in Chinese subjects, in Xinjiang, China.

Although development of CAS is likely to occur among members of a family [20], and several studies have reported relationships between gene and CAS [21–23], knowledge of the role of genomic predictive markers in CAS remains elusive. CAS appears to be active and involves a chronic inflammatory infiltrate with lipid and lipoprotein deposition, including apoB and LDL [4, 5, 8]. It has been suggested that CAS is a manifestation of generalized atherosclerosis, with similar pathogenesis and common risk factors. Whether the initiation of CAS has a genetic contribution as atherosclerosis is unknown. The first investigation of the association of gene polymorphisms with CAS was carried out in 2001 by Ortlepp et al. [24]. They hypothesized that the gene encoding vitamin D receptor (VDR) may correlate with CAS and identified that the B allele of rs1544410 is a risk factor. Several studies have demonstrated that not only clinical atherosclerotic risk factors but also genetic predisposition, are required for the genesis of CAS [20, 22, 25].

In previous studies, we found that the apoB gene was associated with IMT, LDL level, and ABI, which is involved in atherosclerosis and CAS [9]. Similarly, apoB gene polymorphisms may also be a significant independent predictor of CAS. Research on the relationship between polymorphisms of apoB gene and CAS was first reported by Avakian et al., who demonstrated that the X + X+ genotype polymorphism of the apoB gene (rs693) may be a risk factor for CAS (24.0% in cases, 6% in controls, *P* = 0.00) in the Brazilian population [22]. Interestingly, both large-scale case-control study and genome-wide association studies (GWAS) for lipid levels recently reported that the rs693 polymorphism was associated with cholesterol and triglyceride levels, which were risk factors for CAS in European populations [26, 27]. However, in a comprehensive replication study carried out by Gaudreault et al. in French Canadians, the rs693 polymorphism was demonstrated to have no correlation with CAS [20]. In contrast, two other SNPs (rs1042031 and rs6725189) of the apoB gene were significantly associated with CAS [20]. From the above example, we have considered that the association between apoB gene and CAS was different on account of polymorphisms of the apoB gene and ethnic differences.

In the present study, we genotyped polymorphisms of rs693 and rs6725189 SNPs in the apoB gene and found associations with CAS. We found that the rs693 CT/TT genotype and the rs6725189 GT genotype have a higher frequency in CAS patients than that in controls. After adjustments for several confounders, this association remained exist, indicating that the rs693 CT/TT genotype and the rs6725189 GT genotype were independent risk factors for CAS and the risk of CAS was increased in the subjects with the T allele in rs693 and rs6725189. Furthermore, we investigated the relationships between rs693 and rs6725189 and the plasma TC, TG, HDL-C and LDL-C levels. We found that T allele (CT + TT) carriers in rs693 have higher levels of TC when compared to T allele non-carriers. These results might provide convincing evidence for assuming subjects who carry T allele in rs693 may have higher probabilities of suffering from CAS than that who carry C allele.

It remains a challenge to describe the mechanisms that link apoB genetic polymorphisms to CAS. ApoB gene polymorphisms have been suggested to be associated with atherosclerosis [9, 13, 14]. A likely explanation for this may be as follows: first, the various apoB genotypes are associated with divergent effects on plasma lipids: apoB and twin studies suggest that 50–60% of the variation in plasma levels of apoB is genetically determined [11]; second, apoB has been demonstrated to be a better estimation of the total number of atherogenic particles (including LDL, IDL, VLDL, chylomicrons, and chylomicron remnants) than LDL-C, as each atherogenic lipoprotein particle contains one apoB-100 molecule [9, 28–30]; finally, several studies have shown that atherosclerosis may be more directly related to the total number of circulating atherogenic particles entering the arterial wall instead of the concentration of cholesterol in LDL particles, which were widely used for screening to identify individuals at risk for atherosclerosis [9, 31, 32]. Due to the similarity between CAS and atherosclerosis, the apoB gene may also effect the initiation of CAS.

Despite the promising findings in this study, some inherent limitations of this case-control study must be noted. First of all, due to the transversal character of the present study, we failed to get a cause-and-effect relationship between risk factors and CAS. Secondly, the sample size is small and large-scale studies are needed to verify these findings. Finally, the present study lacked functional validation. Additional studies need to be undertaken to clarify the underlying molecular mechanism that associates the apoB gene polymorphisms with CAS.

## Conclusion

This study revealed that both rs693 and rs6725189 of the apoB gene are associated with CAS in Chinese subjects. Subjects with TT/CT genotype or T allele of rs693 and TT genotype or T allele of rs6725189 were associated with an increased risk of CAS. The TT/CT genotypes of rs693 were also associated with increased serum TC levels.

## Abbreviations

apoB: Apolipoprotein B
GWAS: Genome-wide association studies
HDL-C: High density lipoprotein
IDL: Intermediate-density lipoprotein
LDL-C: Low density lipoprotein
SNPs: Single-nucleotide polymorphisms
TC: Total cholesterol
TG: Triglyceride
VDR: Vitamin D receptor
VLDL: Very low-density lipoprotein
